# Repellency and acaricidal efficacy of a new combination of fipronil and permethrin against *Ixodes ricinus* and *Rhipicephalus sanguineus* ticks on dogs

**DOI:** 10.1186/s13071-015-1150-5

**Published:** 2015-10-13

**Authors:** Pascal Dumont, Julian Liebenberg, Frederic Beugnet, Becky Fankhauser

**Affiliations:** Merial SAS, 29 Av Tony Garnier, 69007 Lyon, France; ClinVet International (Pty) Ltd, PO Box 11186, 9321 Universitas, South Africa; Merial Limited, 3239 Satellite Blvd., Duluth, GA 30096 USA

**Keywords:** Ticks, *Ixodes ricinus*, *Rhipicephalus sanguineus*, Permethrin, Fipronil, Dog, Acaricide, Repellent, Frontline Tri-Act®/Frontect®

## Abstract

**Background:**

A blinded, controlled laboratory study was conducted to assess the repellency and acaricidal activity of a topical spot on formulation, a combination of fipronil and permethrin, against *Ixodes ricinus* and *Rhipicephalus sanguineus* ticks on dogs.

**Methods:**

A group of 16 adult mixed breed dogs were randomly divided into treatment and control groups based on pre-treatment live tick counts. On Day 0, the topical spot on formulation of fipronil + permethrin (commercialized under the name Frontline Tri-Act®/Frontect®) was administered to dogs in the treatment group at the minimum recommended dose of 0.1 mL/kg, corresponding to 6.76 mg fipronil/kg and 50.48 mg/kg permethrin. Tick infestations were performed with *I. ricinus* (50 females, 50 males) and *R. sanguineus* (25 females, 25 males) on each dog on Days 2, 7, 14, 21, and 28. Dogs were sedated prior to exposure and confined to crates for approximately 4 h following tick challenge. Ticks were released next to the sedated dogs and tick counts were performed at 4 h and 24 h after the start of exposure for tick counts and removal.

**Results:**

Repellency at 4 h against *I. ricinus* was 72.6, 96.3, 92.8, 89.0, and 88.7 % on Days 2, 7, 14, 21, and 28, respectively. Repellency was 100 % 24 h after exposures on Days 2, 7, and 14 and 99.6 % after exposures on Days 21 and 28. For *R. sanguineus,* repellency at 4 h was 78.0, 96.8, 91.5, 88.0, and 56.8 % on Days 2, 7, 14, 21, and 28, respectively. Repellency at 24 h was 98.6, 100, 98.7, 96.1, and 95.1 % for exposures on Days 2, 7, 14, 21, and 28, respectively.

For *I. ricinus*, acaricidal efficacy recorded at 4 h was ≥ 91.1 % during the full month and was ≥ 99.5 % for the full month when counted at 24 h. Acaricidal efficacy against *R. sanguineus* was ≥ 94.7 % at 4 h from Day 2 to Day 21 and was 71.4 % on Day 28. Acaricidal efficacy at 24 h, was > 97.7 % during the month. Tick counts were statistically significantly reduced in treated dogs at all time-points during the study.

**Conclusions:**

A combination of fipronil and permethrin was highly effective at rapidly repelling and killing both *I. ricinus* and *R. sanguineus* ticks on dogs for at least 4 weeks, with a significant effect at 4 and 24 h after tick exposure.

## Background

Ticks are among the most common external parasites of dogs. Besides the irritation and itchiness they cause to the dogs, they have the potential to transmit pathogenic agents to both dogs and their owners. *Ixodes ricinus* is the main vector of tick-borne encephalitis virus and also transmits the causative agent of Lyme borreliosis [1]. *Ixodes ricinus* is expanding in range and period of activity during the year, possibly as a result of climate change [2]. *Rhipicephalus sanguineus* has a world-wide distribution and is also a common tick species in Europe. Like *I. ricinus*, it can also transmit a variety of pathogens to dogs, including *Babesia* and *Ehrlichia* [3, 4]. It is also capable of passing pathogens from animals to humans causing diseases such as *Rickettsia conori*, the agent of Mediterranean spotted fever [5]. Preventing disease transmission is an important concern to dog owners [6].

Tick repellency should be differentiated from repellency *sensu stricto,* which applies to flying insects [7]. Rather than a fly away effect due to volatile effect of the repellent molecules, repellency against ticks is mainly due to an irritant contact effect and/or a behavioural message to prevent attachment and to leave the host [7]. Authors have proposed to use the term repellency *sensu lato* in the case of irritant effect. Concerning the design of studies assessing repellency against ticks, two possibilities have been suggested: to count ticks that are observed off the dogs (in crates) and to count ticks that are present on dogs after a defined time. The latter is easier under experimental condition. Counting ticks on dogs at early time-points and comparing to control dogs is an indicator of the number of ticks that never climbed on dogs or that fall off due to irritant effect, which is the definition of repellency *sensu lato*. Usually, to simulate a natural infestation, ticks are released first around the animals and are not directly put onto them. In the published literature, the evaluation of repellency is reported at a range of different intervals after exposure. The European Medicine Agency guideline requests a count of ticks on the animals 24 h after exposure, which seems quite long, while other guidelines recommend earlier time points like 4 h after exposure, which seems more accurate [7, 8].

Fipronil has been used as topical spot on for dogs and cats to kills fleas and ticks since the mid 90’s. Combinations of fipronil and various other anti-parasitic compounds are also available [6, 9]. Pyrethroid compounds are known for their ability to repel ticks and flying insects, therefore a combination of fipronil and the pyrethroid permethrin can provide both repellency and acaricidal efficacy against ticks [10]. This study was conducted to assess the repellent and acaricidal effects of Frontline Tri-Act®/Frontect®, a combination of 6.76 % w/v fipronil and 50.48 % w/v permethrin, against *I. ricinus* and *R. sanguineus* on dogs.

## Methods

This report describes an experimental controlled study in dogs conducted in accordance with Good Clinical Practices (GCP) as described in International Cooperation on Harmonisation of Technical Requirements for Registration of Veterinary Medicinal Products (VICH). It was a parallel group, randomized, blinded, controlled efficacy study, conducted following the standard methods for evaluating the efficacy of parasiticides for the treatment, prevention and control of tick infestations [8].

### Animals

Sixteen healthy mixed breed dogs, weighing 10.4 kg to 19.4 kg were studied. None had been exposed to ectoparasiticides for at least 12 weeks. On Day -4, the dogs were randomly allocated to the treatment and control groups based on pre-treatment live attached tick (*R. sanguineus*) counts performed at 24 h after exposure. The dogs were individually caged in an indoor animal unit with an environmentally controlled temperature (15.8 °C to 22.7 °C). Contact between dogs was precluded for the duration of the study. Animals were handled in compliance with the Merial Ethical Committee standards and in compliance with the South African National Standard “SANS 10386:2008 The care and use of animals for scientific purposes”. The dogs were observed for general health conditions daily throughout the study. In addition, all dogs were observed hourly for 4 h following administration of the treatment on Day 0.

### Allocation and treatment

Dogs were ranked by pre-treatment live attached *R. sanguineus* tick counts within sex. They were then randomly allocated to one of two groups. Animals in Group 1 (*n* = 8) served as control dogs. Dogs in Group 2 were treated on Day 0 with the new combination at the recommended minimum dose of 6.76 mg /kg fipronil and 50.48 mg/kg permethrin, according to label instructions.

### Tick infestations and counts

Tick infestations were performed with laboratory-bred European unfed adult *I. ricinus* (50 females, 50 males) and *R. sanguineus* (25 females, 25 males) on each dog on Days 2, 7, 14, 21, and 28. Dogs were sedated and placed into individual crates prior to exposure. The ticks were then released on the floor of the crate, half along the dorsal line of the dog and half along the abdomen, and the dogs remained in the crates for 4 h following introduction of the ticks.

*In situ* tick counts by palpation and visual observation of ticks onto the dogs were performed at 4 h after the start of exposure. At 24 h all dogs were combed to ensure that all ticks were removed and counted. Ticks were categorized as live/dead and free/attached in accordance with the World Association for the Advancement of Veterinary Parasitology (WAAVP) guidelines [8].

### Data analysis

Geometric means were calculated using the tick (count + 1) data and one (1) was subsequently subtracted from the result to obtain a meaningful value for the geometric mean of each group.

For *I. ricinus*, only females were included in the calculations following WAAVP guidelines in accordance to particular biology of this species (only females attach to the dogs), while both females and males were taken into account for *R. sanguineus*. The primary criterion was the assessment of the repellent effect. This was calculated based on the total number of ticks on dogs at 4 and 24 h post-exposure.

Percent Repellency was calculated following European Medicine Agency guideline (EMEA/CVMP/005/2000 – Rev.2) as follows:

Repellency (%) against ticks = 100 × (M_c_ – M_t_) / M_c_, where: 

M_c_ = Geometric mean number of ticks (free or attached, dead or alive) on dogs in the control group at each time point.

M_t_ = Geometric mean number of ticks on dogs in the treated group at each time point.

Acaricidal efficacy was calculated at 4 and 24 h post exposure, based on live ticks only. Percent acaricidal efficacy was calculated as follows:

Acaricidal efficacy (%) against ticks = 100 × (M_c_ – M_t_) / M_c_, where:

M_c_ = Geometric mean number of live ticks (free & attached) on dogs in the control group at each time point.

M_t_ = Geometric mean number of live ticks on dogs in the treated group at each time point.

SAS Version 9.3 TS Level 1 M2 was used for all statistical analyses. For the primary comparisons, groups were compared at each time point by a one-way ANOVA on the logarithmic transformation of (count + 1) data. The testing was 2-sided, with a significance level of 5 %.

## Results

No adverse health effects related to the treatment occurred during the study.

The geometric mean number of *R. sanguineus* ticks on the dogs in the untreated control group ranged from 30.6 to 34.9 at 24 h after exposure, indicating a vigorous tick challenge on all assessment days. Statistically significantly (*p* < 0.001) fewer ticks were recorded for the treated group compared to the control dogs on all assessment time points.

**Fig. 1:**
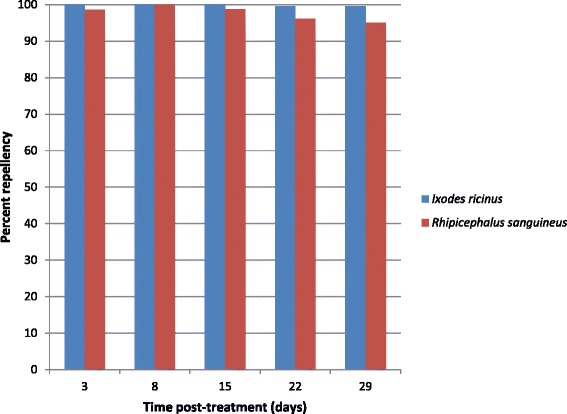
Percent tick repellency following a single topical treatment with a novel combination of fipronil and permethrin. Percent repellency against *Ixodes ricinus* and *Rhipicephalus sanguineus* ticks was based on geometric mean counts at 24 h

Percent repellency against *R. sanguineus* ticks at 4 h after tick exposure was 78, 96.8, 91.5, 88, and 56.8 % on Days 2, 7, 14, 21, and 28, respectively. At 24 h, repellency was 98.6, 100, 98.7, 96.1, and 95.1 % on Days 3, 8, 15, 22, and 29, respectively (Fig. 1, Table 1).

**Table 1:** Analysis of tick repellency (*Rhipicephalus sanguineus)* based on geometric means

4-h assessments				24-h assessments			
Day	Control mean	Treated dogs Mean (Repellency %)	*p*-value	Day	Control mean	Treated dogs Mean (Repellency %)	*p*-value
Day 2	32.7	7.2 (78.0 %)	<.0001	Day 3	30.6	0.4 (98.6 %)	<.0001
Day 7	34.2	1.1 (96.8 %)	<.0001	Day 8	30.6	0.0 (100.0 %)	<.0001
Day 14	33.5	2.8 (91.5 %)	<.0001	Day 15	34.9	0.5 (98.7 %)	<.0001
Day 21	33.5	4.0 (88.0 %)	<.0001	Day 22	34.1	1.3 (96.1 %)	<.0001
Day 28	34.6	15.0 (56.8 %)	<.0001	Day 29	32.3	1.6 (95.1 %)	<.0001

Group 1: Negative control

Group 2: Dogs were treated with Frontline Tri-Act®

The geometric mean number of *I. ricinus* ticks present in the untreated control group ranged from 10.5 to 24.5 at 24 h after exposure, indicating a robust tick challenge. Statistically significantly (*p* < 0.01) fewer ticks were recorded for the treated group compared to the controls on all assessment days.

Percent repellency against *I. ricinus* ticks at 4 h after tick exposure was 72.6, 96.3, 92.8, 89, and 88.7 % on Days 2, 7, 14, 21, and 28, respectively. At 24 h, repellency was 100, 100, 100, 99.6, and 99.6 % on Days 3, 8, 15, 22, and 29, respectively (Fig. 1, Table 2).

**Table 2:** Analysis of tick repellency (*Ixodes ricinus*) based on geometric means

4-h assessments				24-h assessments			
Day	Control mean	Treated dogs Mean (Repellency %)	*p*-value	Day	Control mean	Treated dogs Mean (Repellency %)	*p*-value
Day 2	14.3	3.9 (72.6 %)	0.0094	Day 3	13.1	0.0 (100.0 %)	<.0001
Day 7	13.2	0.5 (96.3 %)	<.0001	Day 8	10.5	0.0 (100.0 %)	<.0001
Day 14	21.1	1.5 (92.8 %)	<.0001	Day 15	19.8	0.0 (100.0 %)	<.0001
Day 21	19.3	2.1 (89.0 %)	<.0001	Day 22	20.3	0.1 (99.6 %)	<.0001
Day 28	24.2	2.7 (88.7 %)	<.0001	Day 29	24.5	0.1 (99.6 %)	<.0001

Group 1: Negative control

Group 2: Dogs were treated with Frontline Tri-Act®

Acaricidal efficacy against both *R. sanguineus* and *I. ricinus* started at 4 h after tick exposure (Tables 3 and 4). Acaricidal efficacy against *R. sanguineus* was ≥ 94.7 % at 4 h after exposure from Day 2 to Day 21 and was 71.4 % on Day 28. Acaricidal efficacy at 24 h was 100 % after exposure on Days 2, 7, and 14 and 98.9 % and 97.7 % after exposure on Days 21 and 28 (Table 3). For *I. ricinus*, acaricidal efficacy observed at 4 h after exposure was ≥ 91.1 % during the full month and was 100 % 24 h after exposure on Days 2, 7, and 14 and ≥99.5 % for exposure on Days 21 and 28 (Table 4). Tick counts were statistically significantly (*p* < 0.01) reduced in treated dogs at all time-points.

**Table 3:** Analysis of acaricidal efficacy (*Rhipicephalus sanguineus*) based on geometric means

4-h assessments				24-h assessments			
Day	Control mean	Treated dogs Mean (Efficacy %)	*p*-value	Day	Control mean	Treated dogs (Efficacy %)	*p*-value
Day 2	32.7	1.6 (95.1 %)	<.0001	Day 3	27.9	0.0 (100.0 %)	<.0001
Day 7	33.8	0.1 (99.7 %)	<.0001	Day 8	30.2	0.0 (100.0 %)	<.0001
Day 14	33.5	1.5 (95.6 %)	<.0001	Day 15	29.3	0.0 (100.0 %)	<.0001
Day 21	32.7	1.7 (94.7 %)	<.0001	Day 22	32.5	0.4 (98.9 %)	<.0001
Day 28	34.4	9.8 (71.4 %)	<.0001	Day 29	30.9	0.7 (97.7 %)	<.0001

Group 1: Negative control

Group 2: Dogs were treated with Frontline Tri-Act®

**Table 4:** Analysis of acaricidal efficacy (*Ixodes ricinus*) based on geometric means

4-h assessments				24-h assessments			
Day	Control mean	Treated dogs Mean (Efficacy %)	*p*-value	Day	Control mean	Treated dogs Mean (Efficacy %)	*p*-value
Day 2	14.2	1.3 (91.1 %)	<.0001	Day 3	13.1	0.0 (100.0 %)	<.0001
Day 7	13.2	0.2 (98.6 %)	<.0001	Day 8	10.3	0.0 (100.0 %)	<.0001
Day 14	20.4	1.0 (95.3 %)	<.0001	Day 15	17.4	0.0 (100.0 %)	<.0001
Day 21	19.3	1.3 (93.2 %)	<.0001	Day 22	19.3	0.1 (99.5 %)	<.0001
Day 28	24.2	1.6 (93.2 %)	<.0001	Day 29	24.1	0.1 (99.6 %)	<.0001

Group 1: Negative control

Group 2: Dogs were treated with IVP with Frontline Tri-Act®

## Discussion

This study demonstrated that a combination of fipronil and permethrin has both a robust repellent effect and a highly efficacious acaricidal effect against two of the most common tick species in Europe, *I. ricinus* and *R. sanguineus*. These effects were evident 2 days after a single treatment, and persisted throughout the 4-week study. The results complement a similarly-designed study that reported on the repellency and acaricidal effect against *Dermacentor reticulatus,* in which repellency observed at 24 h post-exposure was 83.9, 96.5, 95.5, 89.7 and 93.7 % on Days 1, 7, 14, 21 and 28, respectively [11]. These results are also comparable to similar experiments using the same combination product against the same tick species, but with acaricidal efficacy calculated at 48 h [12]. In these studies [12], 48 h efficacy against *R. sanguineus* (2 experiments) ranged from 94.4 to 100 % during the month, and 48 h efficacy against *I. ricinus* ranged from 99.2 to 100 %.

Based on the tick counts recorded throughout the study on the untreated dogs, this study met the guideline recommendations as an adequate test of the treatment [7, 8]. In contrast, there were very few ticks found on the treated dogs (means of 0 to 0.1 for *I. ricinus* and 0 to 1.6 for *R. sanguineus*), confirming the high efficacy.

Repellency has been defined in the EU “Guideline for the testing and evaluation of the efficacy of antiparasitic substances for the treatment and prevention of tick and flea infestation in dogs and cats” (EMEA/CVMP/005/2000 – Rev.2) as, “no tick will attach to the animal or no tick will be detectable on the animal after 24 h following administration of the product.” The assessment of repellency at 24 h seems late to the authors, and many publications prefer earlier counts, usually at 4 h, this earlier timepoint was also measured in this study.

At 24 h, counting ticks (live + dead) present on the animals is most probably assessing repellency and acaricidal efficacy together, whereas counting ticks present at 4 h gives a better view of repellency itself. At 4 h, the repellency effect remained above 72.6 and 56.8 % for *Ixodes* and *Rhipicephalus* ticks, respectively. Between 4 to 24 h, most of the remaining ticks fall off as demonstrated by the efficacy percent observed at 24 h (99.6 and 95.1 % for *Ixodes* and *Rhipicephalus*, respectively). This quick and sustained repellency is a key fact in order to reduce the risk of transmission of tick borne pathogens.

The 24-h acaricidal efficacies against both *I. ricinus* and *R. sanguineus* remained above 97.7 % during the month, which is above the EU guideline (EMEA/CVMP/005/2000 – Rev.2) threshold of 90 % at 48 h. The combination permethrin-fipronil product killed *I. ricinus* ticks within 4 h of exposure with efficacy >91.1 % for the full month. The product demonstrated a sustained speed of kill against *R. sanguineus* ticks at 24 h with efficacy ≥97.7 % for a full month. This is the first time that a topical acaricidal product has been shown to provide such rapid prophylactic speed of kill against *I. ricinus*.

In addition to the quick and sustained repellency, the sustained rapid killing effect is important in order to reduce the risk of transmission of pathogens by ticks. It also reduces the possibility for dog owners to see attached and engorging ticks on their dogs [13].

The high repellent and killing effect observed during this study are most probably due to the additional effect of both permethrin and fipronil, which could be directly compared to permethrin or fipronil alone in further experiments.

## Conclusions

A novel combination of fipronil and permethrin was highly effective at rapidly repelling and killing both *I. ricinus* and *R. sanguineus* ticks on dogs for at least 4 weeks.
